# Lithæmia and Psora

**Published:** 1895-11

**Authors:** Edward Cranch

**Affiliations:** Erie, Pa.


### LITHÆMIA AND PSORA.

Edward Cranch, Ph. B., M. D., Erie, Pa.

(Abstract of a paper read before the County Society, October 2d, 1895.)

The President, Dr. Wilson, having requested a paper on Lithæmia, or some similar subject, one was prepared, comparing the requested subject with Psora, as Hahnemann knew it. Lithæmia was pronounced, on the strength of modern authorities, identical with latent gout, or the gouty diathesis, also called arthritism.

Foster's first definition of psora, viz., scabies, is only a modern limitation of a more general former meaning given by Foster, as, "any cutaneous disease, attended with abundant exudation, pustulation, and crusting," the name derived from ψαν, to rub. Foster might have added the psora "of Hahnemann," and defined it as follows: An acquired or inherited non-venereal contagious diathesis, predisposing the organism to yield readily to all other causes of disease; its local sign, if any, being a vesicular eruption, with furious itching and an alleged specific odor. For its symptoms, when comparatively latent, see Hahnemann's work on *Chronic Diseases* (Vol. I, pp. 58 to 100, 2d ed., 1835). Hahnemann also used the ancient meaning of sycosis, from σῦχογ, a fig, hence the figwart disease. Foster gives this meaning, but the modern meaning is more common, and different, viz., a scab of the beard, or barber's itch. Hahnemann uses miasm in the sense of diathesis, as when he speaks of the gigantic miasm of psora, asserting its derivation from the leprosy, although the quotations from the Old Testament which he offers (incorrectly copied in Hempel's translation) show as great distinction between leprosy and itch then as now. A brief account of his idea of psora is given in § 80 of *The Organon*, where, after speaking of syphilis and sycosis, he says: "Immeasurably greater and more important than both these known chronic miasms is that of psora. While the other two declare their specific inner disorders, one by the venereal chancre, the other by the cauliflower excrescences, psora, after it has completely poisoned the whole interior of the organism with its monstrous miasm, shows itself by means of a peculiar skin eruption, sometimes only in single pimples, with an intolerable tickling and voluptuous itching, having also a specific odor. This psora is the true fundamental cause and promoter of all the other, yes, countless forms of disease, which appear in pathology as distinct independent diseases, under the names of neurasthenia, hysteria, hypochondria, mania, epilepsy, rickets, caries, cancer, neoplasms, gout, hemorrhoids, pruritus ani, bleedings from stomach, nose, lungs, bladder and uterus, asthma, hemicrania, dropsy, paralysis, scrofula, consumption, chronic catarrh, etc., etc." (Hahnemann names many more, here and in his *Chronic Diseases*, but these are enough to show the drift of his argument.)

It is known that Hahnemann was well acquainted with the itch mite, and at one time advocated its extinction by a wash of Hepar-sulphuris and Cream of Tartar.

This was in 1792. In 1805 he began publicly to reject the common practices of his day, in 1810 he published the first edition of his *Organon*, in 1811 he began his *Materia Medica Pura*, in 1816 he began a systematic study of chronic diseases, which he first published in 1828, inserting an allusion to it in a note to § 73 of the fourth edition of his *Organon*, being § 80 of the fifth, and quoted just above.

In the first volume of his *Chronic Diseases* (pp. 22–40, 2d ed.), he gives scores of quotations, all carefully annotated, from writers who observed ill effects following suppression of various skin diseases, and from these quotations he draws his great argument, in the following words (p. 41, *ibid.*): "Who now, after reflection upon these four examples, which are taken from the writings of older physicians, and augmented by my own experience, could well remain so foolish as to overlook that great evil, psora, concealed in the interior, whose signs are the itch-eruption, *and other forms*, scald-head, milk-crust, eczema, etc., and not see that where these local symptoms are outwardly soothed there remains an interior, uncured disease of the whole organism? Who, after reading only these few cases, would deny that psora is, as said above, the most pernicious of all chronic miasms? Who would be so bold as to maintain, with the modern allœopathic physicians, that the itch-eruption, scald-head, and eczema are only superficial skin affections, and can and must, without hesitation, be driven away, while the interior of the body takes no share in them, and so remains healthy? Truly, beyond all mischiefs which one can point out among the modern physicians of the other school, this is the most harmful, infamous, and inexcusable! He who will not, from such and countless other examples, see the contrary of that assertion, beguiles himself on purpose, and works intentionally for the destruction of mankind."

Hering, in his preface to the *Chronic Diseases*, says: "Improvement in diseases takes place from within outward."

Now in modern writings, especially in the works of Henry M. Lyman, of Chicago, writing in Pepper's *Text-book*, and Stedman's *Twentieth Century Practice*, gout is demonstrated to be the underlying diathesis for a list of ailments nearly as long as Hahnemann's that *he* charges to psora. Lyman speaks of "the two great diatheses, gout and scrofula," and declares his inability always to distinguish them. It begins to look as if the old-school men were on the track of psora, but did not know what to call it.

Perhaps they will discover that beyond or within the chemical changes of lithæmia, lack of oxidation, etc., is a morphologic change in cells, inherited or acquired, a sort of *cyto-stenosis*, or cell-contraction, which, by hindering processes of elimination and repair, performs the part that Hahnemann ascribes to psora.

"And so the whirligig of time brings in its revenges."

Lyman and others assert the diathesis of gout to be permanent; Hahnemann was in hopes of curing psora, but, perhaps, it was a glimpse of its real permanence that led him to pen these lines in the fifth edition of his *Organon* (they are not in the fourth): "A human healing art for the normalizing of these countless abnormalities which are often induced by the allœopathic non-healing art does not and cannot exist." As a matter of fact, the very high potencies often go deeper than the 30th or 50th, which Hahnemann used. Hygiene and diet will, if judicious, bring comfort and safety to many a gouty or psoric family. Vaccination appears to be a potent factor in the spread of this diathesis. Tuberculosis, being contagious, is evidently a sort of a graft upon it, as are other infections, which are all worse and harder to treat in the gouty or psoric or cyto-stenotic subject.

In treatment allœopathy wavers between alkalies and acids, mineral waters, lithia, and gin. Colchicum it is afraid of, because it does not comprehend its homœopathic action.

Hahnemann, himself non-psoric, proved about fifty "anti-psoric" remedies that developed in him, for the time, symptoms resembling those of which he gives forty pages in *The Chronic Diseases*, due, he says, to latent psora, most of them found in lithæmia, or gout, also. The following are a few of those symptoms : " Frequent distentions of the abdomen ; frequent asthma ; hands cold, palms sweaty or burning ; muscles easily strained ; chilblains ; night-sweats ; cracking of joints ; tired on waking ; vertigo ; gums bleed easily ; lips swell, especially the upper one ; dread of labor ; irritability ; despondency."

It is evident that the physician should be well acquainted with these symptoms of chronic disease, as given by Hahnemann, not so that he will fall into the routine of calling everything gout, or psora, or cytostenosis, and dosing from a ready-made list without study, but rather that he may be guided in the closer fitting of the case to the remedy.

He must bear in mind Hahnemann's three great warnings, first, not to suppose the doses recommended by him too small ; second, not to use a remedy unless its symptoms correspond with those of the case in hand ; and, third, not to let the remedy be changed or repeated before its action is exhausted.

We must have confidence in our remedies ; we must find out what they can do, alone and uninterrupted, and not interfered with by repetition or alternation. A course of study in this direction, never giving any patient more than one dose of medicine, by itself, at the start, and bravely daring to wait on that remedy as long as it will act ; such a course of study brings the best results, and a feeling of comfort and security to which the alternater and repeater and mixer will always be a stranger.

In the discussion which followed, cases of enlarged glands, of insanity, of convulsions and death were recalled as having followed the retrocession of various eruptions, but no evil effect had been noted from the killing of the itch mite in uncomplicated itch. Two cases were cited, in which the poisoned rash of Rhus-toxicodendron was communicated from one to another by contagion ; once by a towel, once to a bed-fellow. One case was reported of peritonitis, where Aconite and Bryonia failed, when attention was called to the urine, which was loaded with sand. Berberis tincture, one drop every hour, soon cured the patient.

The point was made that few homœopathic writers mention lithæmia; Hughes gives only Lycopodium and Sepia, for it, in his book; Lilienthal adds to these Berberis and Cantharis, and refers to many others by name only. Jahr also refers to Pulsatilla, Cannabis-sativa, and Sarsaparilla; Potter recommends Potassic-permanganate, Lithium salts, Buchu, Colchicum, and Chimaphila; also Hydrochloric and Lactic acids. Others recommend Causticum, Cinnabasis, Natrum-muriaticum, and Calcarea-fluorica.

In the close of the discussion much emphasis was laid upon the need of hygiene, especially in children; also upon the conclusion that inevitably follows from the study of Hahnemann that strict individualization is the prime necessity of all good practice.

Whatever theories Hahnemann may have advanced, the lesson of fitting the remedy to the individual was paramount to all.

